# Vaping as an alternative to smoking relapse following brief lapse

**DOI:** 10.1111/dar.12876

**Published:** 2018-11-28

**Authors:** Caitlin Notley, Emma Ward, Lynne Dawkins, Richard Holland, Sarah Jakes

**Affiliations:** ^1^ Norwich Medical School University of East Anglia Norwich UK; ^2^ Centre for Addictive Behaviours Research, School of Applied Sciences London South Bank University London UK; ^3^ Leicester Medical School Leicester UK; ^4^ New Nicotine Alliance London UK

**Keywords:** electronic cigarette, vaping, smoking relapse prevention, qualitative

## Abstract

**Background and Aims:**

E‐cigarettes are the most popular aid to quitting smoking in the UK. Although many smokers quit, relapse is common. Historically, the literature has reported strong associations between tobacco smoking lapse and relapse following a quit attempt. This article aims to explore how smoking lapse is experienced by those who vape to quit smoking.

**Design and Methods:**

A purposive sample of 40 UK vapers were matched to a sampling frame from a representative sample of UK quitters. Semi‐structured qualitative interviews were conducted. Data were thematically analysed iteratively situating reported experiences of smoking lapse within narrative descriptions of vaping. Iterative categorization was used as a technique to further explore a subset of data specifically focused on smoking lapse.

**Results:**

Analysis revealed that smoking lapse is perceived qualitatively differently when using e‐cigarettes as compared to past quit attempts. Having the pleasurable alternative of vaping meant that full relapse to smoking was not inevitable. Instead, lapses were perceived as ‘permissive’ or ‘purposive’, intentional and contextualised, or for some as unintentional, with the resulting emotional response negatively reinforcing ongoing tobacco smoking abstinence.

**Discussion and Conclusions:**

Our novel findings suggest that the role of tobacco smoking lapse in relation to relapse status may be theoretically redefined, drawing on data from vapers. These findings question the utility of previous theories of the role of smoking lapse in the relapse process. For ex‐smokers, vaping offers a pleasurable, viable pharmacological, but also social and psychological, substitution option for smoking and potentially powerfully alters the experience and threat of any lapse.

## Introduction

E‐cigarettes are the most popular aid to quitting smoking in the UK 1. There is support for vaping as a harm reduction approach and an alternative to tobacco smoking from UK Medical 2 and Public Health 3, 4 bodies. Although many smokers manage to quit, evidence (primarily predating the widespread use of e‐cigarettes) suggests that many successful quitters relapse over time 5. Most vapers use e‐cigarettes for smoking cessation or reduction 3, but we still have little understanding of how vapers use e‐cigarettes to avoid long‐term smoking relapse. Emergent qualitative evidence from our research has suggested that e‐cigarettes may be important in helping people to maintain long‐term smoking abstinence, by substituting not only the physical but also the psychological and social aspects that ex‐smokers had previously enjoyed about smoking 6. It is also important to understand how those who quit smoking using an e‐cigarette experience tobacco smoking lapse and relapse. This is so that we can develop evidence based advice, guidance or interventions to support those who have quit smoking to stay abstinent in the longer term, as relapsing back to tobacco smoking is so harmful to health 7. In the additional analysis reported in this article, we build on our research findings by undertaking analysis of a distinct and unique data set focused on participant experiences of smoking lapse.

A lapse to tobacco smoking can be defined as a one off incident (a single ‘puff’) 8 or a number of cigarettes smoked on any one occasion. Relapse, according to the Russell standard 9, can be defined as more than five instances of tobacco lapse in the previous 50 weeks following a quit attempt, or a return to continuous smoking 10. However, these definitions may not match user or health professional views, as ex‐smokers may not see a one off ‘slip’ as a lapse 11. Published evidence on relapse to tobacco smoking is primarily quantitative, linking predictors of initial lapse to full smoking relapse 10, 12. A spectrum of ‘risk factors’ encompassing: physical (craving, urges, nicotine dependence 13); psychological (self‐efficacy beliefs 14, motivation 15); individual differences such as education level 16; social (partner support 14) and cultural factors (wider environment supportive of smoking 17), have been correlated with tobacco smoking relapse. This medicalised approach assumes a deficit view of tobacco use, positioning the individual as potentially vulnerable to addiction. Arguably, the concept of addiction itself is morally laden, globally characterised by society as inherently bad. Adopting a medicalised approach to tobacco addiction positions the individual who lapses or relapses as in need of ‘treatment’ for their addiction. However, qualitative studies have suggested that, for some, smoking lapse, and relapse, situated in people's lives, is complex, related to unfolding processes of identity development, and involving the loss and regaining of pleasure. A lapse may therefore be situationally adaptive, particularly from a social perspective 18. Adopting this alternative view positions relapse as more than a health behaviour; the understanding of which may ultimately contribute to harm reduction interventions that are implementable, sustainable, and meaningful to the lived realities of smokers and ex‐smokers.

Theoretical understanding of smoking relapse is underdeveloped and would benefit from updating. There are currently no effective recommended approaches to preventing smoking relapse 5. Marlatt & Gordon's theory of relapse, based on cognitive–behavioural theory 19 has underpinned previously developed relapse prevention interventions. Central to the model is a detailed taxonomy of risk factors that can precipitate or contribute to relapse. Although risk factors are both individual and social, the model takes a primarily individualistic stance. The abstinence violation effect (AVE) suggests that following a cue driven initial lapse to smoking (a ‘violation’ of smoking abstinence), the individual's negative emotional response 20 and the causes to which they attribute the lapse 21, create cognitive dissonance 22, resulting in breakdown of willpower, culminating in full smoking relapse 23. This effect is well evidenced 21, 24, 25. However, the AVE phenomenon has, to our knowledge, never been explored in the context of vaping.

Evidence and theorising regarding the role of tobacco smoking lapse and relapse in the lives of users predates the widespread consumer use of e‐cigarettes in the UK as a quitting aid. We therefore propose that a detailed, qualitative understanding of the role of tobacco lapse and relapse is undertaken in the context of vaping, in order to generate hypotheses and develop new theory. In this article we focused specifically on a subset of data reporting users experiences of tobacco smoking lapse, seeking to address the research question: ‘How is smoking lapse experienced by those who vape to quit smoking?’ A qualitative approach was particularly suitable in this context, where existing theory is outdated in the context of a fast moving consumer market establishing e‐cigarettes as integral to the majority of smoking quit attempts.

## Methods

The analysis reported in this article draws on data gathered as part of a wider study (The ECtra study 6). This was a qualitative interview study taking a critical realist epistemological approach, drawing on principles of constructivist grounded theory 26. The approach prioritises the perspectives of individuals and takes at face value the information divulged during the socially constructed situation of the research interview. The interviews broadly sought to illuminate the subjective experiences of vapers who quit smoking using an e‐cigarette, whether or not they reported having maintained abstinence from smoking or experienced tobacco smoking lapse and/or relapse. Participant smoking and vaping status was self‐reported. Participants self‐reporting having quit smoking using an e‐cigarette were recruited using word of mouth, via self‐referral through advertising in local, national and social media, and snowballing to seek referrals from interviewees. The inclusion criteria for the study was broad, aiming for maximum variation in demographic characteristics of individuals (e.g. gender, age, reported smoking history, reported vaping history).

From eligible referrals, a purposive sample of 40 UK vapers were matched by gender and age to a sampling frame of demographic characteristics from a representative sample of past 12 month UK quitters (as reported in Notley *et al*. 6). Interview guides were constructed taking a narrative approach, asking participants to ‘tell the story’ of their history of tobacco use, previous quit attempts, quitting using an e‐cigarette, patterns and experiences of e‐cigarette use over time, and, for this article, the focus was on detailing any lapses or the circumstances surrounding relapse where this had occurred. Interview guides were piloted with ‘experts by experience’ who advised the research team throughout. The cross‐sectional semi‐structured qualitative interviews were conducted between September 2016 and May 2017.

Participants gave written consent for interviews (face‐to‐face or telephone if preferred). Interviews were conducted by experienced researchers trained in qualitative interviewing techniques. Interviews typically lasted 60–90 min and participants were given a £20 shopping voucher as reimbursement for their time. Audio files of interviews were transcribed verbatim and anonymised. Detailed interviewer/field notes were kept following each interview. Participant codes used to reference quotes refer to participant's gender and age (e.g. ‘F24’ for ‘female aged 24’. Letters were used where age and gender duplicates occurred.).

Data were inductively thematically analysed case by case independently by both CN and EW. Thematic analysis is the most appropriate analysis technique in this context, where we aimed to undertake exploratory inductive analysis to allow issues and themes of importance to arise without imposing a priori hypotheses to the data 27. Where they occurred, lapse experiences were analysed as part of the narrative of the individual trajectory through initiating e‐cigarette use, through to quitting smoking. Themes were discussed and compared across cases to identify meta‐themes. As a second stage of analysis, iterative categorisation (IC) 28 was used as a technique by CN to explore meta‐themes. For the analysis reported in this article, the meta‐theme of lapse to tobacco smoking was the focus of analysis. All instances of thematic coding relating to the experience of lapse to tobacco smoking were extracted and further analysed using IC. IC is a rigorous and transparent qualitative analytical technique, ‘coding on’ and developing analysis from initial thematic coding 28, creating a clear audit trail linking analysis back to raw data. This facilitated the process of interpretation by helping to identify clear patterns and situated significance in the data. IC codes were checked and verified by EW.

Ethical approval for the study was granted from UEA's Faculty of Medicine and Health Sciences Research ethics committee, September 2016.

## Results

Overall sample characteristics are reported in Notley *et al*.^6^. The mean age of participants was 41 years (SD: 14.0, range 21–70). All participants identified as White British or European, 16 were employed in managerial, professional or technical occupations, and 33 were recruited in East Anglia with the remainder located across other parts of England. Four interviews were undertaken over the telephone at the request of the participant. There was no discernible difference in data quality between face to face and telephone interviews. Vaping experience varied from starting 2 weeks before interview, to 7 years. Thirty‐one participants were vaping and abstinent from tobacco (19 had reported lapses), six participants had relapsed (five dual using both tobacco and vaping) and three were no longer using either e‐cigarettes or tobacco.

Iterative categorisation of meta‐themes coded under reported situations of tobacco smoking lapse or relapse resulted in four overarching themes. Within these themes we developed 113 unique codes that encapsulated the meaning of the experiences described during interviews (Appendix S1, Supporting Information). Codes are used as descriptive qualifiers for analysis. Quotations were selected from frequently occurring codes across interviews as best representing the analytical point.

### *Vapers' reports of tobacco smoking lapse situations*

Although most of the sample were currently vaping, reporting tobacco abstinence, nearly half reported either brief or regular lapses to tobacco smoking. Lapses reportedly primarily occurred in social situations, particularly those previously associated with smoking:

‘*Even now I still go outside and smoke at the pub, but it is just when you're standing with other people*’ (F26b).

Situated lapse *reasons* were more complex, drawing very specifically on aspects of vaping that were perceived as having been ineffective, in the moment. For example, brief lapses were attributed to device malfunction:

‘*I think I've had, maybe one cigarette since I've been vaping, and that was because my battery died*’ (F21).

Or were blamed on deficits in individual functioning (forgetting):

‘*…there was some wine involved, it was at a social gathering, ex‐colleague, and there was one lonely smoker in the garden and she said ‘come out with me’, and I didn't have my e‐cigarette, and I'm like “oh, I'll just smoke one of yours*”’ (F36b).

These ‘one off’ lapses were justifiable in terms of device or individual ‘failure’. However, a strong theme that appears novel in relation to vaping, is that many of the sample described instances of purposefully lapsing:

‘*there was part of me that wanted to know if it would be the same or not, and part of me wanted to just sort of, “I'll give it a go and just see what's what”, and because I was in a very confident position, then I didn't feel in the slightest bit at risk of sort of regressing, and I think that's part of what it was actually, it was a sort of, it was a check box to say “yes, that's not a problem*”’ (M58).

This ‘purposive lapse’ was situated as a test of resolve, seeing whether one was able to return to being fully abstinent. For these people the lapse was externalised. In contrast, others demonstrated a residual desire for tobacco smoking, perhaps through positive associations with smoking as a social behaviour, such that they positioned lapse as purposive in the sense of being ‘motivated by naughtiness’, enjoying the secret of a purposive lapse, or flaunting an ability to lapse without craving more and fully relapsing ‘just because I can’:

‘*I was like, I'm just going to have a cigarette, just because I could, but I also felt quite naughty doing it, as I was doing it I was like, “I hope he doesn't come down and see me do this*”’ (M30).

When asked how they responded immediately to a lapse, overwhelmingly reported were feelings of disgust:

‘*I have had cigarettes between, in the last three years and they're the most disgusting things, absolutely awful, you know, just out of curiosity sometimes have a cigarette*’ (F62b).

Particularly, unpleasant smell and taste sensations were described:

*‘it just doesn't taste very nice, it makes you smell, and it's not a particularly pleasant thing to be doing compared to vaporising, it's just so much nicer*’ (M37a).

Immediate reactions were described in these very physical terms, with internalised feelings secondary in the descriptions of immediate reactions:

‘*I've tried one, hated it, it made me feel sick so I can't ever go back…sometimes I've smelt someone else having a cigarette, you know, when I've been outside and I just, and just fleetingly think I wouldn't mind a cigarette, but then I remember having tried a cigarette again, and just how sick it made me feel*’ (F48).

### *Reacting and reflecting*

Reflecting on the lapse beyond the immediate reaction, individuals deployed justifications to protect self‐esteem against the potential attack of ‘failure’ at having lapsed. For example, lapse was minimised in discussion, (‘it didn't really count’), positioned as insignificant, i.e. not leading to relapse:

‘*I mean even on this, I do still have the occasional craving for a cigarette, they are very occasional, it's not something that I'm faced with daily or indeed even weekly, so on that particular occasion there were a group of friends, about half of which smoked… so yes obviously I was joining them, initially started on the vape, but of course there was lots of tobacco around…*’ (M44).

Strong physical and emotional immediate responses to a lapse were experienced by participants as being negatively reinforcing, thus potentially *adaptive* in helping to avoid future lapse situations:

‘*then again in December I tried the same thing and was like, “this isn't working for me to be honest”, so I knew at that point it's like, I'm not going to go back to smoking cigarettes’* (M30).

Critically, and recurring frequently across cases, was ‘permissive lapse’. Participants described how, at times, they deliberately set out to have a smoking lapse, either to permit themselves the pleasure of smoking in a time or situation limited way, in the knowledge that they would continue to vape following the lapse:

‘*I always consider myself a vaper, no I will never be a smoker again, so I know that if I have a fag then I can just cut it and it can be a long time before I‘ll have another one for whatever reason, I'm not worried that it's going to be a routine anymore, cos it's not, I've broken the habit as far as I'm concerned, and I feel confident in that, so that's why I'm not afraid if I do feel I need one in extreme circumstances, I will have one, and I won't relapse*’ (F34).

### *Maintaining abstinence from smoking following lapses*

On the path to maintaining abstinence from tobacco smoking, barriers, controls and strategies were discussed at a number of interacting levels. At the individual level, beliefs were fundamental. For some this meant not allowing permissive lapses:

‘*I think if I smoked again I would just be straight back into it*’ (F26b).

Here an ‘abstinence violation discourse’ was apparent. Participants had previous experiences of lapsing during past quit attempts where they had not used e‐cigarettes, which was usually quickly followed by full relapse. Drawing on these past experiences to develop beliefs about current or future likely outcomes helped some individuals to remain abstinent:

‘*….(in the past)… it would just sort of be the occasional cigarette, and then it would be a couple more, and then it would be back on ten a day, so it was just like, that would just be how it went, so it would just go straight back down that slippery slope*’ (M36a).

When discussing smoking abstinence, vapers in our sample talked about the pleasurable and enjoyable aspects of vaping that in many ways had made it easy to stop smoking, certainly compared with previous quit attempts. Vaping was seen as an ‘alternative to smoking’, a direct substitution:

‘*they're the perfect replication of smoking, nothing else gives you that, and if you're like me, and there is a lot of people like me enjoy smoking, the action of it, the feel of it, it becomes, it's important to you isn't it? that feeling, and you enjoy it…it is all about the action and that's the only thing I've ever found that replicates it*’ (F34).

Some people described how they were completely satisfied by vaping:

‘*buying that e‐cigarette, I mean I can't explain it, except to say it satisfied me*’ (M63).

Or went further by describing how vaping, for them, was even ‘better than smoking’:

‘*I much prefer vaping that is something that I actually enjoy, and cigarettes I don't’* (F34).

These pleasurable and positive aspects of vaping are completely novel in comparison to other methods of smoking cessation support. For many users, the positive benefits of vaping meant that they felt that this substitution option was protective against the likelihood of tobacco smoking relapse:

‘*so I'd have a cigarette alongside a drink, that e‐cigarette didn't seem to sate that, but that gradually changed over time, so I can now go out and use an e‐cigarette all night*’ (F46a).

For some, pleasurable aspects of vaping contrasted particularly with less pleasurable aspects of smoking, again potentially protecting against the likelihood of relapse. For example, vapers who discussed enjoying the smells and flavours of vape demonstrated greater levels of dislike, or disgust, for the smell of tobacco smoke. Here, a participant describes the pleasure and enjoyment of flavours as ‘a thing in and of itself’—a uniquely pleasurable aspect of e‐cigarette use that offers something beyond merely smoking cessation:

‘*I really like them, I like the flavours of the liquids, cos that was one thing when I started on like getting flavoured liquids, I was like I really want one that tastes like tobacco, and then I was like, no, I really don't, you can get strawberry and vanilla and yummy things, so I love the flavours… so I really enjoy it, I like it as a thing in and of itself*’ (F38).

### *Shifting identity*

Five participants reported ‘dual using’ both e‐cigarettes and tobacco daily. Dual users discussed feeling under no pressure to quit tobacco smoking, meaning that they could ‘take it or leave it’. While this may have the discursive effect of justifying continued smoking, alternatively the ‘no pressure’ situation may encourage smokers to eventually quit, even if this had not been their intention.

‘*after a while, because I thought it didn't really matter, and potentially had some health benefits of not having any nicotine at all, and I started vaping more, and then sort of gradually I felt I'll have the vape and won't spend money on cigarettes, not only because I don't really crave it that much anymore, and sort of, this is gradually, it wasn't a conscious effort, it was more a by‐product of having a vape that I stopped smoking, because I wasn't planning on stopping, it just happened*’ (M21).

This suggests that time limited dual using when switching to vaping can be a stepping stone towards smoking cessation. A period of dual using, if preceding eventual tobacco smoking abstinence, can be understood less as a phenomenon of physical addiction, and more as shifting social identity. Although many of our sample saw themselves as ‘non‐smokers’, many also retained a smoker identity, as evidenced by a residual attraction for smoking, and the associated psychological and social perceived positive aspects of tobacco smoking:

‘*part of me did miss it, as I said before, I've never disliked smoking per se, I mean, I dislike the effects of smoking, it's always been a sort of nice thing to get that sort of, after you get lunch or whatever, and there's a certain kind of vibe feeling it gives you*’ (F36b).

For some, there was evidence of identity ‘in transition’:

‘*I don't think that I've ever thought that I would ever go back and smoke like I used to… I do get a little bit concerned sometimes that if I put the e‐cig down, if they stopped, if they became suddenly illegal and were banned, then I don't know how I would be in terms of, if I would go back smoking or whether I would go and find another alternative*’ (F33).

Here the participant discusses a shifting identity from smoker to vaper, but draws on a retained identity that is in transition. Significantly she draws attention to cultural and political contexts permitting e‐cigarette use, suggesting that restrictions could impact on subsequent alternative behaviours.

These people we defined as ‘sliders’ towards tobacco smoking abstinence rather than ‘switchers’. However, others within our sample saw themselves very firmly as ‘vapers’, with the switch to e‐cigarettes seemingly complete.

## Discussion

Findings demonstrate that ex‐smokers experience vaping as a pleasurable and enjoyable direct substitution for smoking, particularly apparent in circumstances contrasting the experience of tobacco smoking lapse. Aspects specific to vaping, such as smell, sensory pleasure of inhaling vapour, and the action of vaping, may be protective against smoking relapse, as vapers described how they came to prefer vaping over smoking. Lapses to tobacco smoking were commonly reported, particularly in the early phases of quitting smoking by vaping. Lapse was often purposefully sought in order to ‘test resolve’ in remaining smoke free, or lapses were permissible in certain circumstances due to confidence that relapse could be avoided due to the availability of vaping.

In previous literature, a brief lapse has been strongly associated with smoking relapse 29. In our data too, individuals frequently described how previous instances of lapse prior to vaping inevitably led to relapse. However, in the context of quitting smoking by vaping, brief lapses could be purposive or permissive, without resulting in subsequent relapse. In countries with policies enabling vaping, vaping is a viable, and indeed for many, preferable, alternative to smoking, allowing any sense of ‘failure’ at lapsing to be minimised. By interrupting the almost automatic processes between lapse and relapse through offering an alternative; our exploratory study suggests that relapse need not be inevitable following a brief tobacco lapse amongst those who vape.

The concept of ‘permissive lapse’ implies a ‘no pressure’ solution to forgiving the occasional ‘slip’. This may reduce the pressure that gradually constrains individuals when attempting to maintain abstinence from smoking. This idea underpins the AVE hypothesis. The build‐up of pressure due to cognitive dissonance (the inconsistency between one's resolve to be abstinent and the smoking lapse, along with the internal attribution (‘I'm a failure because I have lapsed’)) results in full‐blown relapse. In the context of continuing to vape, however, individuals seemed able to release some of this pressure. A permissive lapse allows small indiscretions presumably creating less cognitive dissonance, which in turn meant that full relapse was not inevitable.

Strong physical and emotional immediate responses to a smoking lapse, particularly when contrasted with the positive sensory experience of vaping, were experienced by participants as negatively reinforcing. Previous literature on smoking relapse has reported the common phenomenon of negative affect following relapse, creating cognitive dissonance 21. However, the strong disgust reaction described by our sample was primarily sensory—a reaction to dislike of the taste, smell and sensation of tobacco smoking once habituated to the alternative of vaping. In this sense, while vaping, a brief tobacco lapse could be hypothesised as being functionally adaptive in helping to avoid future lapse situations. This is a novel initial finding, as it suggests that, as opposed to a lapse strongly predicting future relapse, lapses may actually negatively reinforce sustained abstinence from tobacco smoking, through initiating a strong ‘disgust’ reaction. This is an important hypothesis arising from and grounded in the analysis of our qualitative cross‐sectional data. Clearly, this may be contested by evidence from other datasets 30, requires future investigation, and particularly testing with longitudinal data.

Previous literature has described ongoing ‘identity work’ as individuals attempt to quit smoking 18, 31, 32. For some, quitting smoking can be exceptionally hard due to the shift in identity that is necessary to prevent smoking relapse. Similarly in our sample, vapers provided evidence of shifts in identity, being ‘in transition’ between a smoker and an ex‐smoker identity, with the alternative identity of ‘vaper’ adopted to a greater or lesser extent. Identity shifts as people move from becoming smokers to vapers require further examination. Particularly for dual users whose identity may be ‘in transition’, there is a need for longitudinal follow‐up to ascertain how transitioning identity unfolds over time, with behaviour either resulting in continued tobacco use or abstinence.

In practice, the findings from our focused qualitative analysis of the experience of vapers quitting tobacco smoking suggest that vaping could be supported in encouraging not just smoking cessation, but long‐term relapse prevention. Particularly, in countries where policies allow vaping, vaping offers a viable alternative to smoking, and is available as a ‘fall back’ option if a brief tobacco smoking lapse occurs. Supporting ongoing vaping then suggests that the UK service approach of encouraging ‘not even a puff’ may need re‐examining, as in fact a brief lapse need not result in relapse if the user has a sufficiently satisfying and enjoyable method of vaping as an alternative. This also has potential implications for the measurement of smoking cessation and relapse prevention outcomes in clinical trials 9.

Theoretically, the concept of the AVE may not apply to those who vape, as our data suggest that brief lapse need not inevitably lead to relapse. However, this conclusion is based on cross‐sectional qualitative findings. This is a limitation—we are able to offer potential hypotheses for future study that require further testing and replication with larger representative populations.

Purposive sampling and rigorous analytical procedures suggest that findings are transferable to wider populations of vapers. However, the sample is qualitative, and thus findings are not generalisable to wider populations of ex‐smokers, since we did not set out to achieve statistical representativeness. Particularly, despite our best efforts, we were unable to sample representatively from lower socioeconomic groups 33. We did not seek biochemical validation of smoking status since our focus was concerned with understanding the participant perspective rather than confirming objective biochemical status. Our sample only included two people who had reported having tried vaping and did not have at least some success in reducing their smoking.

We tentatively suggest that our findings uniquely demonstrate how the role of smoking lapse may be theoretically redefined, in the context of vaping, questioning the utility of previous theories of the role of smoking lapse in the relapse process. For ex‐smokers, vaping appears to offer a powerful substitution option for smoking and an alternative to relapse, even following situations of brief lapse.

## Conflict of Interest

The authors have no conflicts of interest.

## Supporting information


**Appendix S1.** Key analytical themes (IC themes).

**Appendix S2.** Glossary of analytical terms.
