# Supplementary material for: Vaping as an alternative to smoking relapse following brief lapse
Source: Drug Alcohol Rev. 2018 Nov 28;38(1):68–75. doi: 10.1111/dar.12876 (PMC6587865; doi:10.1111/dar.12876)
Supplement: Supplementary file 1 — Appendix S1. Key analytical themes (IC themes). [file DAR-38-68-s001.docx]

**Reported lapse situations**

Lapse at pub (F46b) (F38)

Still lapses at pub (F46b)

Drunk (M37a) (M44) (F46a) (F38)

Lapse with other people (F46b) (M37a) (M49) (F36b) (M40) (M44) (M36a)

Describes self as weak willed (F46b)

Lapse on holiday (M30) (M46) (F33)

Offered cigarette by friend (M30)

Alone but tobacco availability (M30)

Had it in my head I needed nicotine (M40)

**Situated lapse reasons**

Run out of battery (M30) (F21)

Didn’t have vape with me (F36b) (M40)

Purposive (testing) lapse (M30) (M41) (M63) (M46) (F52) (M58) (M36a)

Motivated by naughtiness (M30)

Enjoys secrecy (M30)

‘Just because I can’ (M30) (M37a)

Environmental availability of tobacco (M30) (F36b)

No particular reason (23M) (F34) (F52)

Stress (F21) (F34) (F33) (F38)

‘I needed the nicotine’ (F21) (F34) (F33)

Anger (M49) (F33)

Social cues (M44) (M46) (F48) (F38)

Social exchange of cigarette as object (M44)

**Shared negative buffer**

Advice not to lapse from others experiences (F46b)

**Fear of abstinence violation**

Gone so long without lapse doesn’t want to ‘mess up’ (F46b) (F36b)

Belief that lapse would lead to fast relapse (F46b) (M67) (F36b) (F27)

Past experience of relapse creates fear that it could happen again (F46b) (M36a)

Abstinence best for me (M30) (M67)

Embodied imagining of disgust (M67) (M53) (F48)

Pre-empting relapse – tiredness (F36b)

Pre-empting relapse – lack of willpower (F36b)

Pre-empting relapse – tinnitus (F36b)

Nicotine too addictive (F46b)

Tobacco too accessible (F46b)

**Reported reasons for not lapsing**

Financial (F46b)

I wouldn’t want to do it now anyway (F46b) (M39)

Don’t want to smoke anymore (F46b) (M30) (F33)

Not really tempted (M30) (M63) (F33) (M53)

Anticipation of negative physical reaction if lapsed (F62a)

No lapse (F40) (F62b) (F27) (M63) (F22) (M53) (F36a) (M26) (M70)

Anticipation of self-loathing if lapsed (F62b) (F27)

Respects abstinent self (F62b)

Internalised negative enforcement (F62b)

Learnt alternative coping strategies (F62b)

Vaping as alternative (F62b) (M67) (F34) (M44) (M46) (M58) (M21) (F33) (F46a) (F48) (F36a) (F38) (M39)

Satisfied by vaping (M63) (F34) (M46) (M58) (F38) (M39)

Vaping better than smoking (M37a) (M63) (F34) (M21)

Smoking no longer relevant to me (M39) (M26)

Practical strategies to ensure abstinence (M67) (F46a) (M39)

Back up device (M67) (F46a)

Doesn’t fear withdrawal (M67)

Increased motivation (F36b) (F27)

Doesn’t like smell of smoke (M41) (M63) (M46) (M26)

Health improvement due to not smoking (F33)

Need adequate nicotine substitution (M53)

I love the flavours (F38)

I can vape inside (F38)

**Cultural attitudes to vaping**

Normalisation of tobacco smoking in social group (M30)

**Perceptions of lapse**

Lapse didn’t really count (M30) (M37a)

Permissive lapse (M36b) (M49) (F27) (M63) (F34) (M44) (M46) (F52) (M58) (F33)

Conceivably permissible under high stress conditions (F62b)

**Reactions to lapse - immediate**

Immediate disgust (M30) (F21) (M67) (M41) (M40) (F34) (M46) (M58) (M36a) (F36a)

Enjoyment (23M)

Lack of enjoyment (M37a) (F34)

Shouldn’t be doing this (23M)

Concern about smell (23M) (F21) (M37a)

Dislike taste (M37a) (M40) (F34) (M36a)

Ambivalence (23M)

Feeling sick (F21) (F48)

Guilt (F36b)

Distraction (F36b)

**Reactions to lapse – reflective**

Negative reinforcement (M30) (M37a) (M40) (M46) (M58)

Testing lapse as confirmation of new non-smoker identity (M30)

Guilt at identity role model failure (F34)

Suspended relapse (M44) (F33) (F38)

Minimising importance of lapse – won’t lead to relapse (M44) (F38)

Partial lapse (M36a)

**Dual Use**

Time limited dual use prior to abstinence (M30) (F22) (F46a) (M36a)

Cannabis perpetuates dual using (M30) (M37b)

Vaping encourages switch to vaping cannabis (M30)

No pressure to quit (M49) (M21) (F38)

Purposive (situated) dual use (M40) (M44) (M21) (F22) (F38)

Take it or leave it (F38)

Smoking as a treat (F38)

**Current tobacco status**

Fiercely states abstinence (M30) (M67)

**Identity**

Non smoker (M30) (23M) (F40) (M37a) (M40) (M63) (M63) (M58) (F59) (F33) (M53) (M36a) (M39)

Cannabis culture (M30)

Vaper(F40) (F62b) (F21) (F34)

Retained smoker identity (F21) (M49) (F36b) (M46) (F46a) (F38)

In transition (M67) (F22) (F33) (F38)

Slider not switcher (M49) (M58) (M21)

Different habit (M41)

Friends don’t smoke anymore (F62a) a ‘natural’ smoker (F62a)

Smoked half a cigarette only (M30) (F21)

All of my friends smoke tobacco (M30) (F38)

Friends don’t understand vaping (M30)

Residual desire to smoke (F62a)

No lapses (M67) (F60) (F27) (M63) (F59) (M53)

**The ‘art and craft’ of smoking** (F52)

Loss – rolling a cigarette (M67) (M58)

Loss – cigar at Christmas (M67)

Loss – pleasure (F36b)

Smoking nostalgia (M21)

Social de-normalisation of smoking (M63)

Pleasure of vaping (F34)

Behavioural substitution (F34) (M58)

Policy impacting individual behaviour (F34) (F33)

Wouldn’t want to increase nicotine as prevention of lapse (M44)

Plans to quit e cigarette (M44)

Addiction as inherently bad discourse (M44) (M21)

Vaping as a crutch (M44) (M58)

Experimentation with devices to find the right one (M46) (F22) (M36a) (F38)

Nicotine pixies (M46) (F59) (F48)

Harm minimisation discourse (F52)

Accidental cessation by vaping (M58) (M21) (F38)

No end point to vaping (F38)

Interviews with no lapse data coded:

M22– dual user – was abstinent then relapsed with dual use (cost of e-cig and not being able to tolerate it) straight away – no opportunity for lapse

F29 – relapsed participant – e-cigs have not been successful, straight to relapse – no opportunity for lapse – lapse not discussed

F24 – dual user – has never manager to quit completely – no opportunity for lapse

M25 – dual user –has never managed to quit completely using e-cig – no opportunity for lapse

F25 – abstinent from vaping and tobacco - Tried e-cigs, couldn’t tolerate them, relapsed, then was successful using nicotine replacement therapy – no opportunity for lapse
