# Supplementary material for: Vaping as an alternative to smoking relapse following brief lapse
Source: Drug Alcohol Rev. 2018 Nov 28;38(1):68–75. doi: 10.1111/dar.12876 (PMC6587865; doi:10.1111/dar.12876)
Supplement: Supplementary file 2 — Appendix S2. Glossary of analytical terms. [file DAR-38-68-s002.docx]

**Vaping as an alternative to smoking relapse following brief**

**Additional supplementary appendix: Glossary of analytical terms**

Critical realist epistemological – A ‘middle ground’ approach within the qualitative research paradigm, taking the position that the reality of individual’s lives can only be illuminated through the interpretative lens of the perspective of the researcher.

Constructivist grounded theory – An approach to the development of theory through qualitative analysis allowing both an inductive (purely data driven) and deductive or subjective (guided by existing knowledge, concerns and interpretations of the researcher) approach to data analysis.

Exploratory inductive analysis – Analysis that is not hypothesis drive, but approaches data open to ‘discovering’ potential hypotheses within the data. The inductive approach involves ‘open’ coding of data as it emerges, working upwards towards generating hypotheses.

Iterative categorization - A rigorous and transparent qualitative analytical technique, involving ‘coding on’ and developing analysis from initial thematic coding. Initial open coding generates themes and meta-themes that can then be ‘coded on’ to develop new insights. Iterative categorization creates a clear audit trail linking analysis back to raw data.

Meta-themes – The aggregation of themes and open codes into larger, all-encompassing themes that encapsulate smaller themes.
